# Lower limb rehabilitation in chronic incomplete SCI: a randomized controlled trial protocol comparing combined TMS-tSCS neuromodulation vs. tSCS alone

**DOI:** 10.3389/fnins.2026.1773917

**Published:** 2026-07-07

**Authors:** Ravi Shankar, Gobinathan Chandran

**Affiliations:** 1 Clinical Research and Innovation Office, Tan Tock Seng Hospital, National Healthcare Group, Singapore, Singapore; 2 Department of Medicine, Division of Rehabilitation Medicine, National University Hospital, Singapore, Singapore; 3 Department of Rehabilitation, Alexandra Hospital, National University Health System, Singapore, Singapore

**Keywords:** lower extremity motor score, motor rehabilitation, neuromodulation, spinal cord injury, transcranial magnetic stimulation, transcutaneous spinal cord stimulation

## Abstract

**Background:**

Spinal cord injury (SCI) affects 15.4 million people worldwide, with a substantial proportion of incomplete SCI patients remaining non-ambulatory, highlighting the importance of motor function recovery and mobility in rehabilitation. While transcutaneous spinal cord stimulation (tSCS) has emerged as a promising non-invasive neuromodulation technique for enhancing motor recovery, the therapeutic potential of combining tSCS with transcranial magnetic stimulation (TMS) remains largely unexplored. This combination may leverage the complementary mechanisms of supraspinal and spinal neuromodulation to enhance corticospinal tract plasticity and functional motor outcomes.

**Objective:**

To evaluate the efficacy and safety of combined TMS-tSCS intervention compared to tSCS alone for improving lower extremity motor function in individuals with chronic incomplete spinal cord injury.

**Methods:**

This prospective, randomized, controlled, assessor-blinded clinical trial will enroll 60 participants with chronic (>12 months post-injury) incomplete spinal cord injury (AIS C or D) aged 18–65 years from Alexandra Hospital, Singapore. Participants will be randomized 1:1 to receive either combined TMS-tSCS (intervention group) or tSCS with sham TMS (control group) for 16 weeks (32 sessions). The primary outcome is change in Lower Extremity Motor Score (LEMS) from baseline to 16 weeks. Secondary outcomes include walking speed (10-Meter Walk Test), functional independence (Spinal Cord Independence Measure-III), spasticity (Modified Ashworth Scale), electromyography of the lower limb muscles and neurophysiological measures of corticospinal excitability.

**Expected outcomes:**

We hypothesize that combined TMS-tSCS will yield superior improvements in LEMS (≥2 points greater improvement) compared to tSCS alone, with enhanced corticospinal tract plasticity as evidenced by neurophysiological measures.

**Clinical trial registration:**

ClinicalTrials.gov, identifier: NCT07595497.

## 1 Introduction

### 1.1 Background and rationale

Spinal cord injury (SCI) is a neurological disorder that disrupts motor, sensory, and autonomic functions, with about 15.4 million people affected worldwide as of 2021 (World Health Organization, 2024). Despite the retention of some neural pathways below the level of injury, a substantial proportion of patients with incomplete SCI remain non-ambulatory or dependent on mobility aids in the chronic phase (van Silfhout et al., 2017; van Hedel, 2009), making gait recovery a key rehabilitation priority (Anderson, 2004). The preservation of some neural pathways in incomplete SCI (iSCI) provides a biological substrate for interventions aimed at enhancing neuroplasticity and functional recovery. Among the most disabling consequences of iSCI is lower limb motor impairment, which fundamentally affects mobility, independence, and quality of life (Ahuja et al., 2017).

Traditional rehabilitation approaches for iSCI, including physical therapy, occupational therapy, and locomotor training, remain the cornerstone of post-injury care, although recovery trajectories vary substantially depending on injury severity, completeness, and individual neurological profile (Scivoletto et al., 2004; Burns et al., 2017). While these interventions remain essential components of comprehensive SCI rehabilitation, neuromodulation is increasingly being integrated alongside conventional therapy in specialist SCI rehabilitation centers. Published evidence demonstrates that tSCS delivered concurrently with task-specific activities such as assisted stepping, standing exercises, and gait training can augment the neuroplastic potential of preserved neural circuits and produce functional gains beyond those achieved with conventional therapy alone (Tefertiller et al., 2022; Comino-Suárez et al., 2025). Neuromodulation approaches aim to augment endogenous repair mechanisms by modulating neuronal excitability and promoting activity-dependent plasticity within residual corticospinal and propriospinal pathways (Taccola et al., 2018).

Transcutaneous spinal cord stimulation (tSCS) has emerged as a particularly promising non-invasive neuromodulation technique for SCI rehabilitation. This approach delivers electrical stimulation through surface electrodes positioned over the spine, targeting the posterior roots of the spinal cord, engaging spinal rhythm-generating networks and enhancing the excitability of motor circuits below the level of injury (Minassian et al., 2016). Seminal work by Gerasimenko et al. demonstrated that multisite tSCS could initiate and modulate locomotor circuitry output and induce coordinated stepping movements in non-injured humans, revealing the capacity of transcutaneous stimulation to engage spinal locomotor networks (Gerasimenko et al., 2015). Subsequent studies have confirmed the feasibility and preliminary efficacy of tSCS for improving motor function, standing ability, and walking parameters in in individuals with neurological conditions; notably, Hofstoetter et al. (2021) demonstrated these effects in individuals with multiple sclerosis, while Taccola et al. (2018) reviewed evidence of tSCS-mediated volitional recovery in SCI (Hofstoetter et al., 2021; Taccola et al., 2018). Recent systematic evidence demonstrates that tSCS modulates both spinal and corticospinal excitability, with studies showing decreased H-reflex amplitudes following alternating current tSCS, and increased motor evoked potential amplitudes following direct current tSCS, indicating enhanced neuroplasticity at multiple levels of the motor system (Tajali et al., 2024).

The mechanisms underlying tSCS-mediated motor recovery are thought to involve multiple physiological processes. First, tSCS may enhance the excitability of spinal interneuronal networks, effectively lowering the threshold for activation of motor neuron pools below the level of injury (Taccola et al., 2018). Second, repeated stimulation may induce long-term potentiation-like changes in spinal circuits, strengthening synaptic connections within preserved neural pathways (Tajali et al., 2024). Third, tSCS may engage spinal interneuronal circuits and rhythm-generating networks by providing tonic excitatory input through posterior root afferents, thereby augmenting motor output in response to step-related sensory feedback (Minassian et al., 2016). Recent evidence demonstrates that combined tSCS with activity-based therapy yields clinically meaningful functional gains. Suggitt et al. demonstrated the safety and real-world feasibility of delivering tSCS combined with activity-based therapy across 120 sessions over approximately one year in a community SCI rehabilitation setting, with no device-related adverse events and acceptable tolerability throughout, supporting the viability of prolonged tSCS protocols outside specialist inpatient centres (Suggitt et al., 2025).

While tSCS primarily targets spinal-level circuitry, the integrity and excitability of supraspinal motor centers also play critical roles in motor recovery following iSCI. The motor cortex undergoes substantial reorganization following SCI, with changes in cortical excitability, motor map representation, and intracortical inhibitory-excitatory balance (Nardone et al., 2013; Freund et al., 2011).

Transcranial magnetic stimulation (TMS), a non-invasive technique that induces electrical currents in cortical tissue through electromagnetic induction, is capable of modulating cortical excitability in a frequency-dependent manner, with high-frequency stimulation generally increasing and low-frequency stimulation decreasing excitability (Rossini et al., 2015). Repetitive TMS (rTMS) protocols have shown promise for augmenting motor function in various neurological conditions, including stroke and multiple sclerosis (Lefaucheur et al., 2020). In the context of SCI, preliminary studies suggest that high-frequency rTMS applied to the motor cortex can enhance corticospinal excitability and may improve motor outcomes when combined with physical rehabilitation (Benito et al., 2012; Kumru et al., 2016; Wang et al., 2023). Recent evidence also supports these findings, with randomized controlled trials demonstrating that intermittent theta burst stimulation (iTBS) combined with conventional physiotherapy significantly improveswalking speed, stride length, and functional independence measures in iSCI populations; specifically, Feng et al. (2023) reported LEMS 21.7 → 32.6 in pilot subacute iSCI RCT, while Shi et al. (2025) showed in prospective study (n=138) that 9-week iTBS gave significantly greater improvements, effective rate 86.96% (Feng et al., 2023; Shi et al., 2025). However, while both Benito et al. (2012) and Kumru et al. (2016) demonstrated significant LEMS improvements with rTMS combined with gait rehabilitation, the benefits of rTMS may be limited without concurrent physical training, as cortical neuromodulation alone cannot fully compensate for impaired corticospinal transmission through damaged spinal pathways (Benito et al., 2012; Kumru et al., 2016).

The theoretical rationale for combining TMS and tSCS rests on the principle of convergent neuromodulation, whereby simultaneous or temporally coordinated stimulation of multiple nodes within the motor system may produce synergistic effects that exceed the sum of individual interventions (Barss et al., 2022). By enhancing both cortical motor drive (via TMS) and spinal circuit excitability (via tSCS), combined stimulation may optimize conditions for activity-dependent plasticity throughout the corticospinal axis. This approach aligns with emerging evidence that multi-level neuromodulation strategies may be necessary to maximize functional recovery in complex neurological conditions (Barss et al., 2022).

Despite the compelling theoretical framework, rigorous clinical evidence evaluating combined TMS-tSCS for lower limb rehabilitation in chronic iSCI remains limited. To our knowledge, no randomized controlled trials have systematically compared the efficacy of combined TMS-tSCS against tSCS alone using standardized motor outcome measures. This gap limits our current understanding of whether combining cortical and spinal neuromodulation offers any advantage over spinal stimulation alone. The present protocol is designed as an initial step toward addressing this question, specifically whether adding TMS to tSCS produces greater improvements in lower extremity motor function than tSCS alone. A full characterization of the therapeutic value of this combined approach will require convergent evidence from multiple trials examining different populations, stimulation parameters, and outcome domains.

### 1.2 Study objectives

The primary objective of this study is to evaluate whether combined TMS-tSCS yields superior improvements in lower extremity motor function, as measured by the Lower Extremity Motor Score (LEMS), compared to tSCS alone in individuals with chronic incomplete spinal cord injury.

Secondary objectives include:

1. To compare changes in walking speed and endurance between treatment groups.
2. To assess differences in functional independence outcomes between groups.
3. To evaluate the effects of combined stimulation on spasticity.
4. To characterize neurophysiological changes in corticospinal excitability.
5. To assess the safety and tolerability of combined TMS-tSCS.

### 1.3 Hypotheses

**Primary Hypothesis:** Participants receiving combined TMS-tSCS will demonstrate significantly greater improvement in LEMS from baseline to 16 weeks compared to participants receiving tSCS with sham TMS, with a hypothesized between-group difference of at least two points.

**Secondary Hypothesis:** Combined TMS-tSCS will produce greater improvements in walking speed, functional independence, and corticospinal excitability, defined as increases in MEP amplitude, decreases in MEP latency, and/or reductions in motor threshold of the tibialis anterior muscle, compared to tSCS alone, with sustained effects at 4-week follow-up.

## 2 Methods

### 2.1 Study design

This is a prospective, parallel-group, randomized, controlled, assessor-blinded clinical trial. The study will be conducted at two academic medical centers in Singapore: National University Hospital (NUH) and Alexandra Hospital. Both sites specialize in spinal cord injury rehabilitation and have established neuromodulation research programs. The trial design adheres to the Standard Protocol Items: Recommendations for Interventional Trials (SPIRIT) guidelines and the Consolidated Standards of Reporting Trials (CONSORT) statement for reporting parallel group randomized trials (Chan et al., 2013).

**Figure 1:**
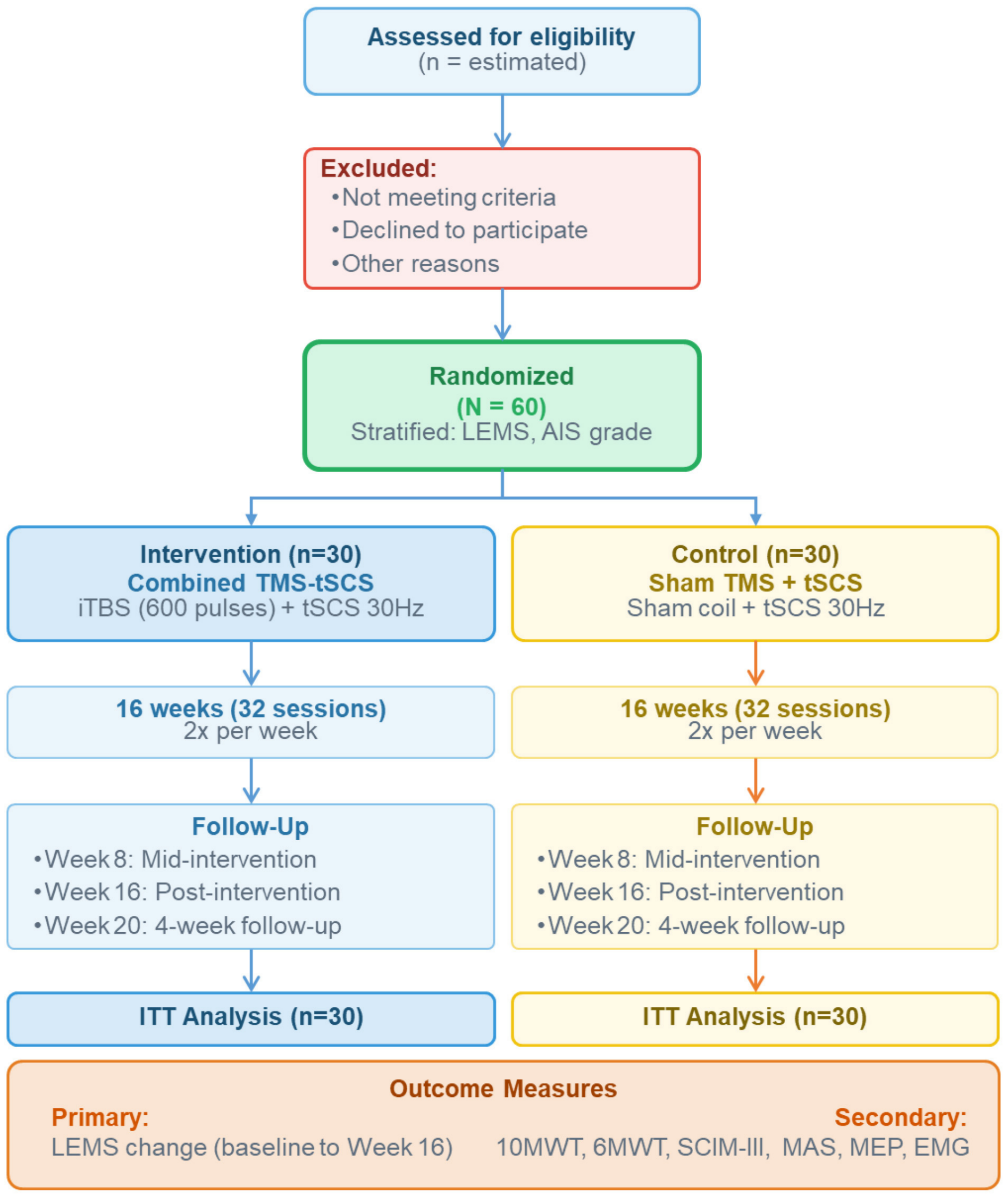
CONSORT flow diagram of the randomized controlled trial comparing combined TMS-tSCS vs. tSCS alone for lower limb rehabilitation in chronic incomplete spinal cord injury. Participants are randomized 1:1 to intervention (combined iTBS + tSCS) or control (sham TMS + tSCS) groups, with assessments at baseline, Week 8, Week 16, and Week 20. AIS, American Spinal Injury Association Impairment Scale; EMG, electromyography; iTBS, intermittent theta burst stimulation; ITT, intention-to-treat; LEMS, Lower Extremity Motor Score; MAS, Modified Ashworth Scale; MEP, motor evoked potential; SCIM-III, Spinal Cord Independence Measure-III; tSCS, transcutaneous spinal cord stimulation; TMS, transcranial magnetic stimulation; 6MWT, 6-Minute Walk Test; 10MWT, 10-Meter Walk Test.

Participants will be randomly allocated in a 1:1 ratio to either the intervention group (combined TMS-tSCS) or the control group (sham TMS plus tSCS). The intervention period will span 16 weeks, with assessments conducted at baseline, 8 weeks (mid-intervention), 16 weeks (post-intervention), and 20 weeks (4-week follow-up; Figure 1). This design allows evaluation of both the immediate effects of the intervention and the durability of any observed benefits.

### 2.2 Study population

Participants will be recruited from the rehabilitation departments at National University Hospital and Alexandra Hospital, Singapore. Recruitment strategies will include physician referrals, review of electronic medical records for potentially eligible patients, and outreach through spinal cord injury support groups and community organizations in Singapore.

A baseline LEMS threshold of >10 was selected to ensure participants retained sufficient residual motor function to engage meaningfully with the rehabilitation protocol and to demonstrate measurable changes on motor and walking outcome measures. This threshold corresponds to at least some volitional movement in multiple lower extremity muscle groups, which is a prerequisite for task-specific training during tSCS sessions (Table 1).

**Table 1:** Eligibility criteria.

Inclusion criteria	Exclusion criteria
1. Age 18–65 years at enrollment	1. History of seizures or epilepsy
2. Chronic traumatic SCI (≥12 months post-injury)	2. Implanted electronic devices (pacemaker, cochlear implant, DBS, and spinal cord stimulator)
3. Incomplete injury (AIS grade C or D)	3. Metallic implants in head or spine
4. Neurological level of injury C2–L1	4. Pregnancy or planned pregnancy
5. Baseline LEMS > 10 points	5. Active psychiatric disorder or cognitive impairment
6. Medically stable	6. Concomitant neurological conditions (stroke, TBI, neuropathy)
7. Able to provide informed consent	7. Skin breakdown at electrode sites
8. Able to commit to full study duration	8. Current participation in another trial
9. Able to attempt the 10-Meter Walk Test and 6-Minute Walk Test with or without assistive devices and standby assistance	9. History of skull surgery or craniotomy
	10. Medications altering cortical excitability (within 2 weeks)

SCI, Spinal Cord Injury; AIS, American Spinal Injury Association Impairment Scale; LEMS, Lower Extremity Motor Score; DBS, Deep Brain Stimulator; TBI, Traumatic Brain Injury.

### 2.3 Sample size calculation

Sample size was calculated based on the primary outcome of change in LEMS from baseline to 16 weeks. Studies of high-frequency rTMS in chronic incomplete SCI have demonstrated LEMS improvements ranging from 1.3 to 4.8 points (Benito et al., 2012; Kumru et al., 2016). Using a conservative estimate of SD = 4.5 points and targeting a clinically meaningful between-group difference of two points (approximately 6% of the total LEMS scale), we calculated the required sample size using a two-tailed independent samples *t*-test with α = 0.05 and power = 0.80.

The calculation yields, *n* = 24 participants per group. Accounting for an anticipated 20% dropout rate based on similar trials in the SCI population, we will enroll 30 participants per group (*N* = 60 total). This sample size also provides adequate power (>0.75) to detect moderate effect sizes (*d* = 0.65) for key secondary outcomes.

### 2.4 Randomization and blinding

Eligible participants will be randomized in a 1:1 ratio to the intervention or control group using a computer-generated randomization sequence with randomly permuted block sizes of 4 and 6. Randomization will be stratified by baseline LEMS (10–30 vs. 31–45) and AIS grade (C vs. D) to ensure balanced distribution of prognostic factors between groups. The randomization sequence will be generated by an independent statistician and concealed using a secure, web-based randomization system accessible only to unblinded study personnel.

Given the nature of the intervention, complete participant blinding is challenging. However, the sham TMS procedure will employ established protocols designed to minimize perceptible differences from active stimulation. A specialized sham coil that produces similar acoustic artifacts and scalp sensations without delivering effective magnetic stimulation will be used for the control group. Participants will be informed that they may receive either active or sham TMS and will be asked not to discuss their perceived treatment assignment with assessors.

All outcome assessors will be blinded to treatment allocation. Assessors will not be present during treatment sessions and will be instructed to avoid discussing treatment details with participants. At the conclusion of the study, assessor blinding will be evaluated by asking assessors to guess participants' group assignments; successful blinding will be indicated by assignment accuracy no better than chance (50%).

The TMS and tSCS interventions will be delivered by trained, unblinded research therapists who will not be involved in outcome assessments. Intervention therapists and outcome assessors will work independently, with no overlap in roles, to maintain assessor blinding.

### 2.5 Interventions

#### 2.5.1 Transcranial magnetic stimulation protocol

For the intervention group, repetitive TMS (rTMS) will be delivered using a 70 mm figure-of-eight coil connected to a MagStim BiStim^2^ magnetic stimulator (MagStim Ltd, Whitland, UK). The device has been registered with the Health Sciences Authority (HSA), Singapore, under Class B medical device notification (CRM-N) for research purposes only. The stimulation target will be the leg motor area of the primary motor cortex, identified using established anatomical landmarks and confirmed by eliciting motor evoked potentials (MEPs) in lower extremity muscles when possible. For participants in whom MEPs cannot be reliably elicited, the target will be defined anatomically as the vertex (Cz) position, located using the international 10–20 system surface measurement landmarks (i.e., the intersection of nasion-to-inion and pre-auricular point-to-point midlines). No EEG recording is required; the 10–20 system is used solely as a standardized method for scalp localization.

The rTMS protocol will employ intermittent theta burst stimulation (iTBS), which has been shown to induce robust and sustained increases in cortical excitability with relatively brief stimulation durations (Huang et al., 2005). The iTBS protocol consists of bursts of three pulses at 50 Hz, repeated at 5 Hz (200 ms between bursts), delivered in 2-s trains with 8-s inter-train intervals. Each session will deliver 600 pulses total over approximately 190 s. Stimulation intensity will be set at 80% of the active motor threshold (AMT) or 80% of maximum stimulator output if AMT cannot be determined. AMT will be determined with the participant maintaining a slight voluntary contraction of the target lower limb muscle (approximately 10%−20% of maximum voluntary contraction, confirmed by visual EMG feedback), and defined as the minimum TMS intensity required to elicit MEPs of at least 200 μV peak-to-peak amplitude in 5 of 10 consecutive trials. Where reliable voluntary contraction cannot be achieved due to motor impairment, resting motor threshold (RMT) will be substituted, as defined in Section 3.2.

For the control group, sham TMS will be delivered using a specialized sham coil positioned identically to the active coil. The sham coil produces acoustic clicks and induces mild scalp sensations through electrical stimulation of scalp electrodes, mimicking the experience of active TMS without delivering effective magnetic stimulation to cortical tissue.

#### 2.5.2 Transcutaneous spinal cord stimulation protocol

Both groups will receive identical tSCS protocols. Stimulation will be delivered using the ARC-EX™ transcutaneous spinal cord stimulation system (ONWARD Medical, Lausanne, Switzerland) through adhesive surface electrodes positioned over the spinous process. The ARC-EX™ device has been registered with the Health Sciences Authority (HSA), Singapore, under Class B medical device notification (CRM-N) for research purposes only. A single cathode electrode (5 cm × 10 cm) will be placed midline over the spinous processes at the vertebral level corresponding to the lumbosacral enlargement (typically T11–L1), consistent with published tSCS configurations for lower limb targeting (Hofstoetter et al., 2018). Two anode electrodes (5 cm × 5 cm each) will be placed bilaterally over the iliac crests.

Stimulation will be delivered as biphasic rectangular pulses at a frequency of 30 Hz, which has been shown to effectively modulate spinal motor circuits (Hofstoetter et al., 2018). The pulse width will be set at 1 ms. Stimulation intensity will be individualized for each participant. The sensory threshold will be defined as the lowest intensity at which the participant reports a tingling or vibrating sensation directly beneath the tSCS electrode on the skin surface. This refers to cutaneous sensation at the electrode site and not referred sensation into the lower limb dermatomes. The motor threshold will be defined as the lowest intensity at which a visible involuntary muscle twitch is observed in the lower extremities. These thresholds will be determined by incrementally increasing stimulation intensity from subthreshold levels. The target tSCS intensity will be set at the highest tolerable level below motor threshold that does not elicit involuntary muscle contractions, typically 40–100 mA depending on individual sensitivity and electrode impedance.

Each tSCS session will last 45 min. During tSCS, participants will engage in structured, task-specific lower limb rehabilitation activities. These may include overground walking practice (with or without assistive devices), standing balance exercises, sit-to-stand transfers, and lower limb strengthening exercises, supervised by a qualified physiotherapist. Activities will be selected and progressed based on the participant's functional level and treatment goals. It is acknowledged that the tSCS electrode configuration and stimulator cables may limit the range of movement-based activities; therefore, therapy tasks will be adapted to accommodate equipment constraints while maximizing active participation. Stimulation parameters (intensity, electrode impedance) will be monitored throughout each session, and adjustments will be made as needed to maintain optimal stimulation delivery.

#### 2.5.3 Combined intervention schedule

Treatment sessions will be conducted on 2 non-consecutive fixed days per week (e.g., Monday and Thursday, or Tuesday and Friday), selected at enrolment based on participant and therapist availability and maintained consistently throughout the 16-week intervention period (32 sessions total). Fixed scheduling was chosen to ensure consistent inter-session intervals for neuroplastic consolidation. Each session will begin with the tSCS (45 min) paired with therapy and followed by the TMS protocol (approximately 3–4 min for iTBS). The temporal proximity of TMS and tSCS is designed to leverage potential synergistic effects of cortical and spinal neuromodulation within overlapping windows of enhanced plasticity.

Sessions will be scheduled with a minimum of 24 h between sessions to allow for neuroplastic consolidation effects and to minimize participant burden. Participants who miss scheduled sessions will be offered make-up sessions within the same week when possible. A minimum attendance rate of 90% (29/32 sessions) will be required for inclusion in the per-protocol analysis.

### 2.6 Concomitant care

Participants will be encouraged to continue their usual rehabilitation activities and physical therapy throughout the study period. To minimize confounding, participants will be asked not to initiate, discontinue, or alter the dose of medications likely to influence outcomes of interest, particularly anti-spasticity agents, anticonvulsants, and centrally acting drugs, unless a change is clinically required. Participants will not be asked to modify any pre-existing medication regimen as a condition of enrolment. Any medication changes occurring during the study will be documented and accounted for in sensitivity analyses. Any changes to medications or rehabilitation programs will be documented and considered in sensitivity analyses. Participants will not be permitted to enroll in other interventional trials or initiate new neuromodulation treatments during the study period.

### 2.7 Study schedule

See Table 2 in study schedule.

**Table 2:** Schedule of enrollment, interventions, and assessments.

Assessment/activity	Screening	Baseline	Week 8	Week 16	Week 20	Ongoing
Informed consent	X					
Eligibility assessment	X					
Medical history	X					
Randomization		X				
tSCS + sham TMS (control group)						2x/week
tSCS + active iTBS TMS (intervention group)						2x/week
ISNCSCI/LEMS		X	X	X	X	
10MWT/6MWT		X	X	X	X	
SCIM-III		X	X	X	X	
Modified Ashworth scale		X	X	X	X	
Electromyography		X	X	X	X	
MEP assessment		X		X	X	
Adverse event monitoring						Each visit

ISNCSCI, International Standards for Neurological Classification of Spinal Cord Injury; LEMS, Lower Extremity Motor Score; 10MWT, 10-Meter Walk Test; 6MWT, 6-Minute Walk Test; SCIM-III, Spinal Cord Independence Measure-III; MEP, Motor Evoked Potential.

## 3 Outcome measures

### 3.1 Primary outcome

The primary outcome measure is change in Lower Extremity Motor Score (LEMS) from baseline to Week 16, the immediate post-intervention timepoint (Table 3). Week 16 was selected as the primary endpoint because it captures the maximal cumulative effect of the combined neuromodulation protocol following completion of all 32 sessions, aligns with established convention in TMS and tSCS rehabilitation trials (Benito et al., 2012; Kumru et al., 2016; Feng et al., 2023; Shi et al., 2025), and minimizes confounding from post-intervention behavioral variability (e.g., changes in physical activity or unsupervised practice) that may differentially affect the two arms during follow-up. Week 20 is retained as a pre-specified secondary time-point to assess durability of treatment effects within the MMRM framework (Section 5.3).

**Table 3:** Summary of outcome measures.

Outcome	Type	Measurement tool	Clinical significance
Lower extremity motor score	Primary	ISNCSCI (0–50 points)	MCID: 2–3 points; predicts ambulation
Walking speed	Secondary	10MWT (m/s)	MCID: 0.15 m/s
Walking endurance	Secondary	6MWT (meters)	MCD: 45.8 meters; MCID estimates range 30–50 meters
Functional independence	Secondary	SCIM-III (0–100)	Comprehensive SCI-specific measure
Quality of life	Secondary	EQ-5D-5L	Health utility index and EQ-VAS; validated in SCI populations
Spasticity	Secondary	Modified Ashworth Scale (0–4)	Clinician-rated muscle tone
Corticospinal excitability	Exploratory	MEP amplitude/latency	Neurophysiological plasticity marker
EMG	Exploratory	EMG activity	Muscle activity/recruitment

ISNCSCI, International Standards for Neurological Classification of Spinal Cord Injury; 10MWT, 10-Meter Walk Test; 6MWT, 6-Minute Walk Test; SCIM-III, Spinal Cord Independence Measure-III; MEP, Motor Evoked Potential; MCID, Minimal Clinically Important Difference.

The LEMS is a component of the International Standards for Neurological Classification of Spinal Cord Injury (ISNCSCI), representing the summed strength of five key muscle groups in each lower extremity (hip flexors, knee extensors, ankle dorsiflexors, great toe extensors, and ankle plantarflexors), graded on a 0–5 scale for each muscle group. The total LEMS ranges from 0 to 50 points, with higher scores indicating greater motor strength (Kirshblum et al., 2011).

The LEMS is a well-established measure of lower extremity motor function in SCI populations, assessed as part of the ISNCSCI examination and standardised across clinical settings (Kirshblum et al., 2011). Importantly, LEMS has been shown to be a strong predictor of ambulatory capacity and functional independence following SCI (Ditunno et al., 2007). LEMS improvements have been associated with meaningful functional gains in chronic iSCI populations, though establishing definitive minimal clinically important difference thresholds remains an area of ongoing investigation (Wu et al., 2015). The measure's strong correlation with walking ability and independence outcomes makes it particularly valuable for assessing motor recovery interventions.

### 3.2 Secondary outcomes

All secondary outcomes will be assessed at baseline, Week 8, Week 16, and Week 20, with the exception of MEP assessments, which will be conducted at baseline, Week 16 and Week 20 only, as shown in Table 2.

**Walking Function:** The 10-Meter Walk Test (10MWT) will assess walking speed over a 10-meter distance at both comfortable and maximum speeds, with excellent reliability (inter-rater *r* = 0.97, intra-rater *r* = 0.98) established for SCI populations (Jackson et al., 2008). The 6-Minute Walk Test (6MWT) will measure walking endurance as the total distance walked in 6 min on a standardized course (Lam et al., 2008). The type and level of assistive device used during baseline assessment of the 10MWT and 6MWT (e.g., rolling walker, forearm crutches, cane, or no device) will be documented and standardized across all subsequent assessment time points to ensure reproducibility and valid within-participant comparisons.

**Functional Independence:** The Spinal Cord Independence Measure-III (SCIM-III) is a disability scale specifically developed for persons with SCI, assessing self-care (0–20 points), respiration and sphincter management (0–40 points), and mobility (0–40 points). The total score ranges from 0 to 100, with higher scores indicating greater independence (Catz et al., 2007).

**Quality of Life:** The EQ-5D-5L (EuroQol Group) will be administered to capture participant-perceived health status across five dimensions: mobility, self-care, usual activities, pain/discomfort, and anxiety/depression, along with a visual analog scale (EQ-VAS) for overall health. The EQ-5D-5L is widely validated in SCI populations, is brief to administer, and generates utility values that can support future health economic evaluations of the intervention (Herdman et al., 2011).

**Spasticity:** The Modified Ashworth Scale (MAS) will be used to assess spasticity in bilateral hip flexors, knee extensors, and ankle plantarflexors, graded from 0 (no increase in tone) to 4 (limb rigid in flexion or extension).

**Neurophysiological Measures:** Motor evoked potentials (MEPs) will be recorded from the tibialis anterior and soleus muscles bilaterally in response to single-pulse TMS over the motor cortex. MEP amplitude (peak-to-peak), MEP latency, and cortical silent period duration will be measured to characterize corticospinal excitability and inhibitory function. Additionally, recruitment curves will be constructed by delivering TMS at intensities ranging from 100% to 150% of motor threshold to assess the input-output properties of corticospinal projections (Rossini et al., 2015).

Motor threshold for neurophysiological assessments is defined as the minimum TMS intensity required to elicit MEPs of ≥50 μV peak-to-peak amplitude in at least 5 out of 10 consecutive trials in the target muscle at rest (resting motor threshold, RMT) or during slight voluntary contraction (active motor threshold, AMT).

**Electromyography (EMG):** Surface electromyography (EMG) will be recorded bilaterally from the tibialis anterior, medial gastrocnemius, rectus femoris, and biceps femoris muscles. Bipolar Ag/AgCl surface electrodes will be placed over the muscle belly in accordance with SENIAM guidelines, with an inter-electrode distance of 20 mm (Hermens et al., 2000). A reference electrode will be placed on the ipsilateral fibular head. Signals will be amplified (gain × 1,000), bandpass filtered (20–500 Hz), and sampled at 2,000 Hz. Data will be acquired using the Noraxon Ultium EMG wireless surface EMG system (Noraxon USA Inc., Scottsdale, AZ, USA) and visualized using Noraxon MR-XP 1.07 software. EMG will be recorded during dedicated assessment sessions at baseline, Week 8, Week 16, and Week 20, and not during treatment sessions. Participants will perform three maximum voluntary isometric contractions of each recorded muscle, each held for 5 s with 60-s rest intervals, to allow normalization of EMG amplitude. Continuous lower limb EMG will also be recorded during the 10MWT at comfortable speed to characterize muscle activation timing and recruitment patterns during gait. Instructions and rest intervals will be standardized across all assessment sessions.

## 4 Safety monitoring

### 4.1 Adverse events

Adverse events (AEs) will be systematically monitored and documented throughout the study period. At each treatment session, participants will be asked about any symptoms or problems experienced since the last visit. All AEs will be recorded on standardized case report forms, including onset date, duration, severity (mild, moderate, and severe), relationship to study intervention (unrelated, possibly related, probably related, and definitely related), and outcome.

Anticipated adverse events associated with TMS include:

1. Transient headache (common, typically mild).
2. Scalp discomfort at stimulation site.
3. Transient tinnitus or hearing changes.
4. Fatigue.
5. Seizure (rare, < 0.1% with established safety guidelines).

Anticipated adverse events associated with tSCS include:

1. Local skin irritation at electrode sites.
2. Transient paresthesias during stimulation.
3. Muscle soreness or cramping.
4. Autonomic dysreflexia (AD), a potentially serious complication in participants with injury levels at or above T6, characterized by acute hypertension, bradycardia, headache, and diaphoresis. All staff will be trained in AD recognition and emergency management, including upright positioning, removal of stimulation, identification and elimination of noxious stimuli, and administration of rapid-acting antihypertensive agents (e.g., sublingual nifedipine) as per institutional AD management protocols. Participants with injury levels at or above T6 will have blood pressure monitored at the start, during, and at the end of each session.

### 4.2 Serious adverse events

Serious adverse events (SAEs), defined as events that result in death, are life-threatening, require hospitalization or prolongation of existing hospitalization, result in persistent or significant disability/incapacity, or are considered medically important, will be reported to the Institutional Review Board (IRB) and Data Safety Monitoring Board (DSMB) within 24 h of the study team becoming aware of the event. Causality assessment will be performed by the principal investigator and verified by an independent physician.

### 4.3 Data safety monitoring board

An independent DSMB will be established to monitor participant safety and study conduct. The DSMB will consist of at least three members with expertise in clinical trials, neurorehabilitation, and biostatistics, none of whom have direct involvement in the study. The DSMB will meet after enrollment of 20, 40, and 60 participants to review safety data and may recommend modifications to the study protocol or early termination if safety concerns arise. Pre-specified stopping rules will be established based on the incidence of serious adverse events. All TMS procedures will adhere to international consensus guidelines for the safe use of transcranial magnetic stimulation (Rossi et al., 2021).

## 5 Statistical analysis

### 5.1 Analysis populations

The intention-to-treat (ITT) population will include all randomized participants, analyzed according to their assigned treatment group regardless of protocol adherence. This will serve as the primary analysis population. The per-protocol (PP) population will include participants who complete at least 80% of scheduled treatment sessions and all primary outcome assessments. The safety population will include all participants who receive at least one treatment session.

### 5.2 Primary analysis

The primary analysis will compare change in LEMS from baseline to Week 16 between treatment groups using analysis of covariance (ANCOVA), with baseline LEMS, AIS grade, neurological level of injury, and study site as covariates. The treatment effect will be estimated as the adjusted mean difference between groups with corresponding 95% confidence interval. Statistical significance will be determined at the two-sided α = 0.05 level.

Missing data for the primary outcome will be handled using multiple imputation under the missing-at-random assumption. Sensitivity analyses will include complete-case analysis, last-observation-carried-forward, and pattern-mixture models to assess the robustness of findings under different missing data mechanisms.

### 5.3 Secondary analyses

Secondary outcomes (10MWT, 6MWT, SCIM-III, MAS) will be analyzed using similar ANCOVA models. For outcomes measured at multiple time points (baseline, Week 8, Week 16, and Week 20), mixed-effects models for repeated measures (MMRM) will be used to characterize the trajectory of change over time and assess treatment-by-time interactions.

Responder analyses will compare the proportion of participants achieving clinically meaningful improvements (≥2 points LEMS, ≥0.1 m/s 10MWT speed) between groups using chi-square tests or Fisher's exact test as appropriate.

### 5.4 Subgroup analyses

Pre-specified subgroup analyses will examine treatment effects according to baseline LEMS (10–30 vs. 31–45), AIS grade (C vs. D), neurological level (cervical vs. thoracic), time since injury (12–36 months vs. >36 months), study site (NUH vs. Alexandra Hospital), and baseline ambulatory status (non-ambulatory vs. ambulatory with assistance). Subgroup analyses will be considered exploratory and interpreted with appropriate caution given the limited sample size for detecting interaction effects. An additional exploratory subgroup analysis will compare treatment effects between participants in whom MEPs are present at baseline vs. those in whom MEPs cannot be reliably elicited. This analysis will explore whether preservation of corticospinal tract conduction is a prerequisite for, or modifier of, treatment response to combined TMS-tSCS.

## 6 Ethical considerations

### 6.1 Regulatory approval

This study will be conducted in accordance with the Declaration of Helsinki (2013 revision), ICH-GCP guidelines, and Singapore's Human Biomedical Research Act (2015). The protocol, informed consent forms, and participant-facing materials will be submitted to the National Healthcare Group Domain Specific Review Board (NHG DSRB) for approval prior to study commencement at both sites. Recruitment will not begin until full ethical approval is granted. Any protocol amendments will be submitted to the NHG DSRB prior to implementation. The trial will be prospectively registered on ClinicalTrials.gov before enrolment of the first participant.

### 6.2 Informed consent

Written informed consent will be obtained from all participants prior to enrolment. For participants with high cervical injuries (C2–C4) who may have limited hand function precluding a written signature, witnessed consent will be permitted, whereby the participant provides verbal consent in the presence of an independent witness who co-signs the consent form. This process will comply with NHG DSRB witnessed consent procedures. The consent process will include detailed explanation of study procedures, potential risks and benefits, alternatives to participation, and the voluntary nature of participation. Participants will be given adequate time to consider the information and ask questions before deciding whether to participate. Consent will be documented using IRB-approved consent forms.

### 6.3 Data management and confidentiality

All study data will be collected and managed using secure electronic data capture systems compliant with Good Clinical Practice (GCP) guidelines. Participant confidentiality will be maintained through use of unique study identifiers, secure data storage with access controls, and de-identification of data for analysis. Study records will be retained for a minimum of 7 years following study completion in accordance with Singapore regulatory requirements.

## 7 Discussion

This protocol describes a rigorous randomized controlled trial designed to evaluate the efficacy of combined TMS-tSCS for enhancing lower extremity motor function in chronic incomplete SCI. If our hypothesis is confirmed, this study will provide the first high-quality evidence supporting the use of combined cortical and spinal neuromodulation for motor rehabilitation in this population.

The theoretical rationale for combining TMS and tSCS is grounded in principles of convergent neuromodulation and Hebbian plasticity. By simultaneously enhancing cortical motor drive and spinal circuit excitability, we aim to optimize conditions for strengthening residual corticospinal connections through activity-dependent mechanisms. The iTBS protocol employed in this study has been shown to induce long-term potentiation-like effects in cortical circuits (Huang et al., 2005), while tSCS at 30 Hz effectively modulates spinal interneuronal networks and may facilitate motor neuron recruitment (Hofstoetter et al., 2018). The temporal proximity of these interventions is designed to ensure that enhanced cortical outputs arrive at spinal targets during windows of heightened excitability, potentially maximizing associative plasticity effects. This approach is supported by precedent from studies combining transcranial direct current stimulation (tDCS) with robot-assisted gait training, where pairing cortical neuromodulation (tDCS) with task-specific locomotor training via robotic gait orthosis has been shown to enhance lower extremity motor function in individuals with incomplete SCI; notably, no spinal or peripheral electrical neuromodulation was involved in that paradigm (Raithatha et al., 2016). Similar principles of timing-dependent associative plasticity have been demonstrated in studies pairing cortical stimulation with peripheral nerve stimulation in other neurological populations, suggesting that the convergence of enhanced descending drive with heightened spinal excitability within overlapping temporal windows may be critical for optimizing activity-dependent plasticity across the corticospinal axis (Lefaucheur et al., 2020).

Several aspects of our study design merit discussion. First, we have chosen LEMS as the primary outcome because it is a well-validated, reliable measure that directly assesses lower extremity motor function and has established relationships with functional outcomes such as ambulation. While walking tests and functional independence measures provide important complementary information, LEMS offers a more direct assessment of the motor system changes targeted by our intervention.

Second, our selection of chronic SCI patients (>12 months post-injury) is intentional. While neuroplasticity may be more robust in the acute and subacute phases following injury, functional gains observed in chronic patients are more likely attributable to the intervention rather than spontaneous recovery. Furthermore, chronic patients represent a population with substantial unmet clinical need, as most rehabilitation resources are concentrated in the early post-injury period. Should this combined intervention demonstrate efficacy, future work will need to address pathways for integrating multi-level neuromodulation into routine outpatient or community-based rehabilitation services, including considerations of equipment access, therapist training, and cost-effectiveness, to ensure that trial-level benefits can be translated into sustainable clinical practice. We acknowledge that a considerable body of SCI neuromodulation research has been conducted in chronic populations; the unmet need we refer to is specifically the limited availability of evidence-based neuromodulation-augmented rehabilitation services in routine clinical care for this group.

Third, the use of sham TMS in the control group allows us to isolate the specific contribution of cortical neuromodulation while controlling for tSCS effects and non-specific factors (attention, time, expectation). The specialized sham coil employed in this study has been validated to produce credible placebo effects while not delivering effective cortical stimulation (Duecker and Sack, 2015).

Potential limitations of this study include the relatively modest sample size, which constrains our ability to detect treatment effects in subgroup analyses. Additionally, while assessor blinding is maintained, participant blinding to TMS is imperfect, potentially introducing expectation effects. We have attempted to minimize this through use of the validated sham procedure and by asking participants not to discuss their perceived treatment assignment.

An important design limitation is the absence of a sham-tSCS control arm or a rehabilitation-only control group. Both arms receive active tSCS, meaning the study cannot isolate the specific contribution of tSCS itself from non-specific or placebo effects. This is particularly relevant given emerging evidence from recent trials suggesting that differences in functional recovery between active tSCS and sham-tSCS groups may be smaller than initially anticipated, as observed in tSCS trials including Comino-Suárez et al. (2025), who found no significant between-group differences at end of intervention period despite follow-up trends favouring tSCS (Kumru et al., 2016). The current two-arm design was chosen pragmatically to focus on the added value of cortical neuromodulation (TMS) over spinal stimulation alone. Future factorial or three-arm designs incorporating a sham-tSCS group and/or a rehabilitation-only control would be needed to fully disentangle the contributions of each intervention component.

If this trial demonstrates superior efficacy of combined TMS-tSCS over tSCS alone, it would support the development of multi-level neuromodulation protocols for SCI rehabilitation and provide a foundation for future studies examining optimal stimulation parameters, treatment duration, and combination with task-specific training. Conversely, a negative result would provide valuable information about the limitations of this approach and redirect research efforts toward alternative strategies.

This prospective, randomized, controlled, assessor-blinded clinical trial will provide rigorous evidence regarding the efficacy of combined TMS-tSCS for improving lower extremity motor function in chronic incomplete SCI. By targeting both cortical and spinal components of the motor system, this combined approach has the potential to produce synergistic effects that exceed those of either intervention alone. The results of this trial will inform clinical practice and guide future research in neuromodulation-based rehabilitation for SCI.
